# Nicotine pouches: a review for the dental team

**DOI:** 10.1038/s41415-023-6383-7

**Published:** 2023-10-27

**Authors:** Joshua M. Jackson, Anthony Weke, Richard Holliday

**Affiliations:** 41415351629001 School of Dental Sciences, Faculty of Medical Sciences, Newcastle University, Newcastle Upon Tyne, UK; Newcastle upon Tyne Hospitals NHS Foundation Trust, Newcastle upon Tyne, UK; 41415351629002 School of Dental Sciences, Faculty of Medical Sciences, Newcastle University, Newcastle Upon Tyne, UK

## Abstract

Nicotine pouches are tobacco-free products that are becoming increasingly popular in the UK. They are held between the user's lip and gum to provide a source of nicotine. This article describes the composition of nicotine pouches, the legality surrounding their production and sale, patterns of use and explores possible oral and general health effects of their usage.

## Key points

- Nicotine pouches, a tobacco-free product, are becoming increasingly popular in the UK and globally.
- Nicotine pouches are likely to be a substantially 'lower-risk product' as compared to tobacco smoking.
- Nicotine pouches may present another option in tobacco harm reduction and smoking cessation.
- The oral health effects from the use of this product are unknown but likely to be in keeping with other orally administered nicotine products. Localised gingival recession might be seen.

## Introduction

**Fig. 1:**
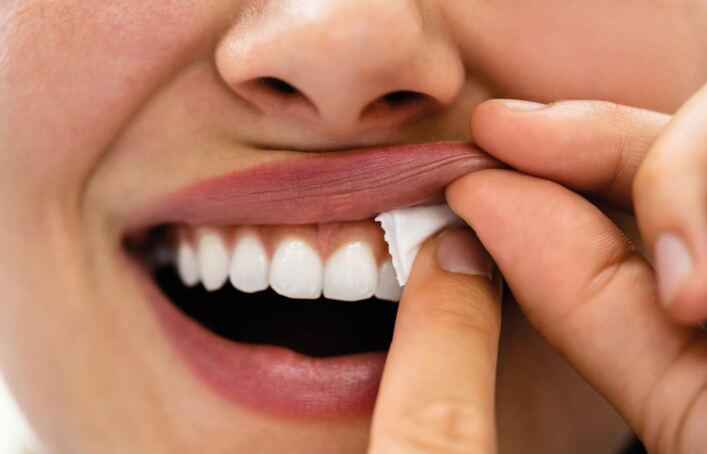
A nicotine pouch being inserted into the bucco-labial sulcus. Image courtesy of iStock.com/AndreyPopov

Oral nicotine pouches are tobacco-free products that are held between the user's lip and gum, as shown in Figure 1. They deliver nicotine through the oral mucosa, being absorbed via mucous membranes and entering the blood stream. Nicotine pouches are of a similar concept to smokeless tobacco products, such as 'snus', which are widely used in countries such as Sweden.

Nicotine pouches provide a source of nicotine but without the main constituent of carcinogen-associated tobacco.^1^^,^^2^Nicotine pouches have becoming increasingly popular in the UK since they entered the market in 2019 and are most prevalent among smokers.^3^ It is of increasing importance for clinicians to have an awareness of such products and their possible implications on oral and general health. This paper will aim to summarise for the dental team the biological, public health, regulatory and clinical aspects of nicotine pouches.

## Origins and composition

Nicotine pouches are a relatively new product that began to predominately be sold in Europe, USA and Japan from around 2019.^4^ Nicotine, a tertiary amine, is the main active ingredient. Once absorbed, it selectively binds to nicotinic cholinergic receptors in the brain, causing the release of dopamine and triggering a pleasurable response.^5^ A significant decrease in brain reward function has been demonstrated with nicotine withdrawal, contributing to its addictive nature.^6^

**Fig. 2:**
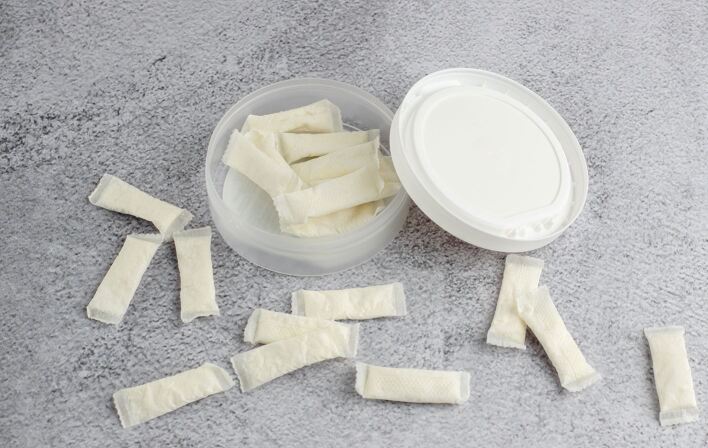
Nicotine pouches presented in a container with approximately 20 pouches. Image courtesy of iStock.com/Oleksandr Shatyrov

In addition to nicotine, approximately 80-90% of a nicotine pouch is made up of water and microcrystalline cellulose contained within a permeable pouch, which acts as the non-tobacco substrate. Other ingredients, such as additives and flavourings, are also present at food-grade standard and are sold in a variety of fruit and other flavours, such as mint and coffee.^4^^,^^7^ Nicotine pouches generally contain artificial sweeteners rather than sugars and so pose little direct risk of the development of dental caries.^8^ Nicotine pouches are readily available across the UK for a relatively small cost of around £5-6.50 per pack as of March 2023.^9^ They are sold in a small container consisting of approximately 20 pouches, as shown in Figure 2.

Total nicotine content within each pouch has been quoted as between 1.29-6.11 mg per pouch, with some 'strong' versions now marketed as around 11 mg/pouch.^9^^,^^10^Comparatively, both nicotine gum and lozenges are prescribed at concentrations between 1 mg and 4 mg depending on the number of cigarettes smoked per day, up to 15 pieces per day.^11^

Chemical analysis of nicotine pouches has shown that they contain much lower toxic compounds than Swedish snus and tobacco smoking.^7^ Furthermore, early data have shown nicotine pouches to be less biologically active than Swedish-style snus products.^12^ Thus, nicotine pouches may provide another alternative nicotine replacement product to aid in smoking cessation, though its long-term effects currently remain inconclusive.

## General health effects

Due to their lack of combustion and absence of tobacco leaf, nicotine pouches are likely to be a substantially 'lower-risk product' relative to tobacco smoking.^4^ However, there is currently a lack of evidence to evaluate the absolute health effects of these specific products.

Nicotine is the main active ingredient and is probably the main reason most people will use these products. Nicotine binds to cholinergic receptors, activating a complex pathway leading to the eventual release of dopamine, glutamine and gamma aminobutyric acid. These contribute to the pleasurable feelings users experience and are also responsible for its addictive properties.^5^ Nicotine is not classed as a carcinogen and has been used for several decades in the form of nicotine replacement therapy (NRT).^13^^,^^14^Nicotine has well-documented cardiovascular effects, but these are likely to pose little risk in most individuals.^15^ There is some debate if nicotine may impact on the developing brain.^16^ Common side effects of NRT use are described in Table 1.

**Table 1:** Common side effects of nicotine replacement therapy

NRT product	Adverse effects reported	Reference
Transdermal patches	Skin irritation and sensitivity	Fiore 1992^29^
Gum	Gastrointestinal disturbances, jaw pain and oro-dental problems	Fiore 1992^29^
		Palmer 1992^30^
Lozenges	Nausea, hiccups	Marsh 2005^31^
Inhalator	Throat irritation, coughing, oral burning	Schneider 1996^32^
Nasal spray	Nasal irritation, runny nose	Hartmann-Boyce 2018^33^
Sublingual tablets	Burning, hiccoughs, mouth ulcers, coughing, burning sensation	Wallstrom 1999^34^

## Oral health effects

At the time of writing, there were no published data investigating the impact of nicotine pouches on oral health and so further research is required in this area. However, the authors can speculate potential impacts based upon our knowledge of the ingredients and of similar products.

Nicotine itself has been used in oral forms (lozenges, gum, sprays) for over 40 years and is our mainstay of smoking cessation support. The World Health Organisation listed NRT as an essential medicine in 2009.^17^ Relatively minor side effects are well-documented and include mouth and throat soreness, mouth ulcers, hiccups and coughing. A systematic review and meta-analysis combined data from hundreds of smoking cessation studies. From observational studies, they reported that 5.4% of orally administered NRT users would experience mouth/throat soreness. Randomised controlled trial data showed that orally administered NRT was associated with both mouth/throat soreness (OR: 1.87; 95% CI: 1.36-2.57) and mouth ulcers (OR: 1.49; 95% CI: 1.05-2.20).^18^ It is important to highlight that mouth ulcers are a common side effect of stopping smoking and reported in about 40% of individuals, although the data presented above accounted for this by comparing to suitable controls.^19^ There is no reported evidence of increased oral disease (cancer, caries, periodontal disease) with orally administered NRT. Numerous laboratory-based studies have investigated the potential effects of nicotine on oral cells as summarised in a recent systematic review.^20^ The review concluded that generally, the evidence was limited and contradictory for a number of outcomes. With respect to cytotoxicity, a dose response was reported and it was suggested that the nicotine level in the saliva of smokeless tobacco users may be high enough to achieve cytotoxicity. This may be of relevance to nicotine pouch users given the similar delivery route.

Interestingly, nicotine has been shown to have angiogenic effects (growing new blood vessels), the opposite to what we observe with tobacco use. Theoretically, this could have wound healing properties, but also, it could also facilitate growth of existing tumours, although this is not supported by clinical data.^21^

Duration of use is an important consideration. Traditional NRT is generally intended for medium-term use with a typical course being three months. Current National Institute for Health and Care Excellence guidance advises that NRT can be used as a complete or partial substitute for tobacco either in the short- or long-term.^11^ A concern is that nicotine pouch users may use the product for much longer periods than most traditional NRT users would, and this may increase the potential for side effects, although data are needed to confirm if this is the case.

We might look towards smokeless tobacco products such as snus to give an indication of potential side effect of a product being held against the oral tissues for prolonged periods. The carcinogenic effects of smokeless tobacco products are not applicable as these are not present in nicotine pouches - nicotine is not a carcinogen.^14^ However, one of the well-known side effects of these products is localised gingival recession near where the product is held, and we might anticipate that we will see similar effects in nicotine pouch users due to their similar methods of administration.^22^^,^^23^

Cariogenic risk is also worthy of consideration. We would anticipate this to be fairly low risk given that all the major manufacturers report their products to contain sweeteners rather than sugars. However, there may be some localised effects on plaque accumulation on the tooth surfaces near where the pouch is held, leading to increased caries risk.

## Could they have a role as a smoking cessation tool?

To the authors' knowledge, there is currently no published evidence involving the use of nicotine pouches as a smoking cessation tool. Nicotine pouches have been shown to deliver nicotine effectively, with maximum observed concentrations of nicotine similar to that of lozenges and higher than nicotine gum.^24^ Subjectively, nicotine pouches were favoured to lozenges with respect to taste and sensations within the mouth.^7^^,^^25^

In comparison to cigarettes, nicotine pouches produced a slower and lower magnitude of nicotine delivery to the user. These characteristics have been suggested to have 'lower abuse liabilities' but were regarded as less satisfying and rewarding.^26^^,^^27^ One small study suggested that when compared with nicotine gum, nicotine pouches have been shown to have lower mean ratings of craving, but this finding was not statistically significant.^25^

Currently, there are several NRT products available within the UK: transdermal patches, gum, lozenges, inhalator, nasal spray and sublingual tablets.^11^^,^^28^ A number of adverse effects have been reported with the use of the currently available NRTs (Table 1), which could lead to patients trying alternative therapies to aid in smoking cessation, such as nicotine pouches. It could be suggested that nicotine pouches are simpler and easier to use than other forms of NRT, such as nicotine gum, which requires a more complex technique to use ('park and chew').^35^

In a recently published survey, only 15.9% of respondents that had experience of smoking or vaping were aware of nicotine pouches.^36^ It seems likely that nicotine pouches would have a similar effect profile to e-cigarettes, although data are needed to confirm this.

Globally, there has been some considerable opposition to the introduction of nicotine pouches and the way in which they are marketed.^37^^,^^38^ It has been suggested that nicotine pouches may introduce the 'gateway effect', providing a steppingstone to cigarette smoking due to the combination of addictive nicotine, pleasant flavours and attractive packaging. This could also be attractive to previous non-smokers or young people.^39^ The 'gateway effect' has also been a concern around e-cigarettes, with much debate and analysis in the tobacco-control literature. A recent paper attempted to triangulate individual- and population-level data and concluded that the 'causal claims about a strong gateway effect from e-cigarettes to smoking are unlikely to hold, while it remains too early to preclude other smaller or opposing effects'.^40^ There is currently a paucity of evidence to determine the prevalence of nicotine pouch usage among young people. According to the International Tobacco Control Youth 2021 survey, 4% of 16-19-year-olds reported ever using nicotine pouches. This considers data across the USA, Canada and England.^41^ When considering England in isolation, 1% of this age group used pouches in 2019.^42^ Further data are urgently required to assess the popularity of these products among young people, who anecdotally appear to be the target of marketing strategies by manufacturers.

## Legislation

Nicotine pouches are currently regulated in the UK by default under the General Product Safety Regulations (GPSR). The Tobacco and Related Product Regulations (TRPR) currently regulates all categories of tobacco products, with parts 6-8 of the TRPR regulating e-cigarettes. However, the TRPR does not cover nicotine pouches. As nicotine pouches are only currently marketed as consumer products in the UK, they do not fall under the Jurisdiction of the Medicines and Healthcare Regulatory Agency (MHRA). E-cigarettes, on the other hand, which have a pathway to be brought into the UK market as either a consumer product or medicinal product, are notified to MHRA before they can be legally sold in the UK. With the increasing use and popularity of nicotine pouches, it is likely that different regulatory stakeholders will begin to consider a focused regulatory framework for nicotine pouches in a similar way to that which they did with e-cigarettes.

In the UK, the use of nicotine pouches among adults more than doubled from 0.14% in November 2020 to 0.32% in October 2021,^3^ and although usage remains low in the UK, it is increasing and drawing the attention of regulatory authorities. For example, in 2021, Action on Smoking and Health (ASH) responded to a call for evidence from the government for UK product safety review, with concerns regarding how nicotine pouches are regulated. Their concerns were that GPSR is not the appropriate regulatory framework for nicotine pouches which are potentially highly addictive and are accessible to children under the GPSR. ASH were also cautious that there are currently no limits on nicotine strength, restrictions on age of sale, and restrictions on advertising, promotion and sponsorship of nicotine pouches.^17^ It is likely that more regulatory stakeholders will get involved to advocate for focused regulation for nicotine pouches as their usage increases in order to minimise the risks of unwanted use by certain populations such as young people.

The UK currently promotes the use of e-cigarettes (a novel nicotine product) for smoking cessation largely due to evidence of its success in this regard and relative safety compared to smoking. Given that nicotine pouches are also a novel nicotine product, will the UK soon begin to look at the prospects of nicotine pouches in smoking cessation? Will this influence future regulation of nicotine pouches?

## Conclusion

Nicotine pouches are a new product that the dental professional should be aware of, particularly in smokers and ex-smokers. They are likely to have a relatively low-risk profile, similar to other forms of orally administered nicotine; however, the prolonged and regular use may give increased risk of local oral problems. Further research is required.
